# An Intriguing Case of Non-typhoidal Salmonella Presenting as Bacteremia and Bilateral Cellulitis in a Diabetic Patient

**DOI:** 10.7759/cureus.80297

**Published:** 2025-03-09

**Authors:** Alexander Von Roenn, Japjit Serai, Christian R Dondonan, Robert Gemayel, Elizabeth Brooks

**Affiliations:** 1 Internal Medicine, McLaren Macomb Hospital, Mount Clemens, USA

**Keywords:** bacteremia, cellulitis, diabetes, non-typhoidal salmonella, salmonellosis

## Abstract

Non-typhoidal *Salmonella *typically presents acutely with gastrointestinal disease/illness. These infections infrequently manifest outside the intestines, often due to bacterial translocation from the gut. However, it is exceedingly rare for it to present as cellulitis, especially in a bilateral distribution. A 35-year-old male with a past medical history of diffuse adiposity, essential hypertension, peripheral neuropathy, and depression presented to the emergency department (ED) for evaluation of sudden onset bilateral lower extremity swelling, pain, and redness. The patient first noted these symptoms upon awakening two days prior, accompanied by night sweats. He mentioned having burned his right medial lower leg on a motorcycle exhaust pipe a week earlier but reported appropriate healing of the burn. Of note, the patient owned a corn snake, a potential vector for *Salmonella*, and was diagnosed with diabetes during this admission. He was ultimately found to have diffuse cellulitis of his bilateral lower extremities, with subsequent blood cultures yielding a diagnosis of bacteremia secondary to salmonellosis.

## Introduction

Salmonellosis is one of the most common foodborne illnesses worldwide. The Centers for Disease Control and Prevention (CDC) estimates that *Salmonella *causes approximately 1.35 million infections and 420 deaths annually in the United States alone [1]. Globally, there are 95 million cases of *Salmonella *infections [2]. The infection is typically acquired through the consumption of contaminated food or water, with poultry, eggs, meat, and unpasteurized dairy products being the most frequently implicated sources [3]. However, *Salmonella *has more direct zoonotic sources as well. Some species such as *Salmonella enterica* and *Salmonella bongori* are found in reptiles [4].

Severe complications of non-typhoidal *Salmonella *include sepsis, osteomyelitis, lung infections, and septic arthritis [5]. While gastrointestinal symptoms like diarrhea and abdominal cramps are the hallmark of salmonellosis, atypical presentations can occur [6]. One such manifestation is cellulitis, a bacterial skin infection that may arise following direct contact with contaminated reptiles, which are natural reservoirs of this pathogen [7]. In cases where skin breakdown occurs, *Salmonella *can enter the bloodstream or surrounding tissue, leading to localized infections characterized by redness, swelling, and pain [8,9]. Though rare, several prior cases of salmonellosis with cellulitis have been documented [9,10].

This case report presents an unusual instance of salmonellosis, in which bilateral lower extremity cellulitis was the primary symptom in a previously healthy middle-aged patient. The report explores the clinical progression and etiology of this unique presentation and discusses the importance of thorough history-taking in uncovering potential sources of infection. 

## Case presentation

A 35-year-old male patient with a past medical history of neuropathy, depression, and hypertension presented to the emergency department (ED) for bilateral lower leg swelling, redness, and pain that had onset two days prior upon awakening. The patient reported night sweats preceding his lower extremity findings, as well as some transient shortness of breath. He denied recent sick contacts, insect or animal bites; however, he had burned his right leg on his motorcycle exhaust one week prior. His social history was significant for ownership of a corn snake, for which he claimed strict adherence to hygienic handling. He specifically denied any direct contact between the snake and his burn wound. The patient also denied a prior history of rashes. The shortness of breath had resolved, and he complained only of diffuse pain in his lower legs.

In the ED, he met two of the four criteria for systemic inflammatory response syndrome (SIRS), elevated heart rate (HR) and white blood cell (WBC) count, with a suspected source, and underwent a full septic workup including the collection of two sets of blood cultures. The patient's presenting vitals were: blood pressure (BP) of 150/92, HR of 99, respiratory rate (RR) of 18, and temperature of 36.7°C. The patient had a WBC count of 16.19. Intravenous ampicillin-sulbactam 1.5 g every eight hours and vancomycin with pharmacy to dose were initiated for broad-spectrum antibiotic coverage. He was also found to be hyperglycemic at 184 (reference range: 70-110 mg/dL) with a hemoglobin A1c (HbA1c) value of 9.6% and was subsequently diagnosed with type-2 diabetes mellitus (DM2). Physical examination was notable for a diffuse erythematous, edematous, and tender rash on the bilateral legs, extending between the ankles and knees. The rash was non-purulent, with poorly defined borders overlying non-pitting edema. The rash was slightly more extensive on the right lower extremity, where the prior burn wound appeared to be healing appropriately. No eschar was present, nor was there any obvious skin breakdown on the left lower extremity. Pulses were intact, and the rest of the physical exam was unremarkable. 

A septic workup yielded blood cultures positive for *Salmonella *twice, which was sensitive to ampicillin, cefotaxime, ceftazidime, ceftriaxone, ciprofloxacin, levofloxacin, and trimethoprim/sulfamethoxazole. Antibiotic coverage was altered with ceftriaxone 2 g every 24 hours in lieu of ampicillin-sulbactam. The patient steadily improved over the next couple of days, with decreasing pain, swelling, and redness in his legs bilaterally. He was discharged home on oral ciprofloxacin 500 mg twice a day and doxycycline 100 mg twice a day for a total antibiotic regimen of 14 days, as recommended by the infectious disease team.

## Discussion

This case highlights a unique presentation of salmonellosis, where cellulitis was the initial symptom. It is suspected that this patient’s burn, while healing appropriately, likely created a localized disruption in the skin barrier, facilitating the translocation of *Salmonella *into the bloodstream via direct contact with skin exposed to physical contact with reptiles, especially in the context of the patient’s poorly controlled diabetes. Hyperglycemia is known to impair immune function and may provide a favorable environment for bacterial proliferation, leading to systemic infections [11]. What remains unclear, however, is how and why the patient developed cellulitis in two distinct locations. We postulate that once bacteremic, the patient’s burn-free leg became infected in the same manner most cellulitis cases occur. Cellulitis is most commonly found in the lower extremities for a variety of reasons. The lower legs are more prone to injuries, abrasions, and breaks in the skin, such as cuts, insect bites, or ulcers, which can serve as entry points for bacteria [12]. Blood flow in the lower extremities is impacted by gravity, especially in individuals with poor venous circulation or edema. This impaired blood flow impedes the delivery of WBCs, nutrients, and oxygen to enable healing and promotes stasis and edema [13]. Taking into consideration this patient’s obesity and new-onset uncontrolled diabetes, it creates an ideal environment for bacterial proliferation.

Reptiles are recognized carriers of various *Salmonella *serotypes, which can be transmitted through direct handling or indirect contact with contaminated environments. It has been reported that up to 50% of "domesticated" snakes may harbor *Salmonella *[7]. This should increase awareness and education for pet owners, especially those with vulnerable populations at home, such as children or immunocompromised individuals. In this case, the patient’s history of handling the pet corn snake, despite proper hygienic measures, likely facilitated the transmission of the bacteria. However, we cannot rule out the possibility that prolonged proximity to a reptile colonized with *Salmonella *may not be sufficient to infect a susceptible human host. There is also research suggesting that humans can host *Salmonella *bacteria in a “non-pathogenic carrier-state” in their gallbladders due to the suppression of virulence factors by bile acids [14]. This hypothesis suggests that, despite recent responsible husbandry practices, less stringent handling of the reptile in the past could have seeded our patient’s gallbladder, with the bacteria lying dormant until recently when the patient’s uncontrolled blood sugars altered his homeostatic balance in favor of bacterial proliferation.

Cellulitis caused by *Salmonella *is less frequently documented compared to more common bacterial pathogens like *Staphylococcus aureus *or *Streptococcus pyogenes*. There are very few cases of *Salmonella *causing cellulitis. A case by Othman et al. highlights cellulitis caused by *Salmonella typhimurium* that led to necrotizing fasciitis [10]. The patient in this case was immunocompromised with no identified source of origin of the *Salmonella typhimurium*. In our case, we were able to identify the possible source of *Salmonella *coming from a snake. The recognition of *Salmonella *as a potential cause of skin infections is vital, as it may lead to delays in appropriate diagnosis and treatment. Had this patient’s HR been only slightly lower, he would not have met SIRS criteria, and blood cultures that ultimately dictated more specific treatment may not have been collected. Consider also the scenario in which this patient, or another similarly infected person with a less common pathogen masquerading as classical cellulitis, presents to an outpatient clinic. It is not farfetched to imagine suboptimal treatment and subsequent progression of disease. This case serves as a stark reminder of how easily anchoring bias can blind physicians and underscores the critical role of detailed history-taking in diagnosing the etiologies of common disease patterns.

## Conclusions

This case report describes a rare presentation of salmonellosis as bilateral cellulitis in a 35-year-old male patient. While non-typhoidal *Salmonella *usually causes gastrointestinal symptoms, factors like a recent burn, reptile exposure, and poorly controlled diabetes likely contributed to bacterial translocation and infection. This highlights the need for thorough patient history and awareness of atypical presentations to ensure timely diagnosis and appropriate treatment. Recognizing *Salmonella *as a potential cause of cellulitis is crucial, especially in patients with unique risk factors.
